# The cerebrospinal fluid proteome of preterm infants predicts neurodevelopmental outcome

**DOI:** 10.3389/fped.2022.921444

**Published:** 2022-07-19

**Authors:** Kristin Leifsdottir, Kerstin Jost, Veronica Siljehav, Eric P. Thelin, Philipp Lassarén, Peter Nilsson, Ásgeir Haraldsson, Staffan Eksborg, Eric Herlenius

**Affiliations:** ^1^Department of Women’s and Children’s Health, Karolinska Institutet, Stockholm, Sweden; ^2^Astrid Lindgren Children’s Hospital, Karolinska University Hospital, Stockholm, Sweden; ^3^The Children’s Hospital of Iceland, Reykjavik, Iceland; ^4^Department of Neurology, Karolinska University Hospital, Stockholm, Sweden; ^5^Department of Clinical Neuroscience, Karolinska Institutet, Stockholm, Sweden; ^6^SciLifeLab, Department of Protein Science, KTH Royal Institute of Technology, Solna, Sweden

**Keywords:** preterm infant, proteomic profile, cerebrospinal fluid, neurodevelopmental outcome, neonatal sepsis, outcome prediction

## Abstract

**Background:**

Survival rate increases for preterm infants, but long-term neurodevelopmental outcome predictors are lacking. Our primary aim was to determine whether a specific proteomic profile in cerebrospinal fluid (CSF) of preterm infants differs from that of term infants and to identify novel biomarkers of neurodevelopmental outcome in preterm infants.

**Methods:**

Twenty-seven preterm infants with median gestational age 27 w + 4 d and ten full-term infants were enrolled prospectively. Protein profiling of CSF were performed utilizing an antibody suspension bead array. The relative levels of 178 unique brain derived proteins and inflammatory mediators, selected from the Human Protein Atlas, were measured.

**Results:**

The CSF protein profile of preterm infants differed from that of term infants. Increased levels of brain specific proteins that are associated with neurodevelopment and neuroinflammatory pathways made up a distinct protein profile in the preterm infants. The most significant differences were seen in proteins involved in neurodevelopmental regulation and synaptic plasticity, as well as components of the innate immune system. Several proteins correlated with favorable outcome in preterm infants at 18–24 months corrected age. Among the proteins that provided strong predictors of outcome were vascular endothelial growth factor C, Neurocan core protein and seizure protein 6, all highly important in normal brain development.

**Conclusion:**

Our data suggest a vulnerability of the preterm brain to postnatal events and that alterations in protein levels may contribute to unfavorable neurodevelopmental outcome.

## Introduction

Despite improvement in the care of preterm infants during the last decades, neurodevelopmental deficiencies remain a major cause of chronic neurological morbidity throughout life (1, 2). The degree of immaturity at birth and continued brain development in the neonatal period, determines long-term neurological outcome of preterm infants (3). Insults during the neonatal period may alter these developmental processes. However, the diagnosis of preterm brain injury and outcome prediction are difficult to perform. Brain abnormalities diagnosed with neuroimaging techniques correlate with adverse outcome, but normal diagnostic findings do not necessarily predict normal neurodevelopment (4). Infants born prematurely have decreased brain volume at term-equivalent age on magnetic resonance imaging (MRI) compared to term infants (5). This might indicate a suboptimal neurodevelopment after preterm birth. Furthermore, alterations in microstructural neural connectivity, not captured on imaging-defined volume measures, are important promoters of disturbed brain development (6, 7). Microstructural white matter disorganization has even been observed in adolescents who were born preterm, correlating with lower scores of language function compared with former term controls (8).

Not only functional and structural brain enriched proteins, but also many immune proteins are present in the brain. They have a role in brain development and synaptic plasticity (9). While inflammatory cascades may become detrimental, inflammatory proteins are part of the host defense system, with beneficial functions. It has also been suggested that inflammation may mediate neuroprotection by preconditioning the developing brain (10). Increased levels of inflammatory proteins in CSF has been correlated with preterm birth (11).

Recently, novel techniques for sensitive protein analysis in body fluids, like antibody-based proteomic targeting, have emerged. These techniques are increasingly utilized for comprehensive examination of CSF, with the purpose of discovering biomarkers of brain pathology (12). Changes in the highly active central nervous system (CNS) are reflected in biochemical alterations in CSF, that is in direct contact with the extracellular matrix of the brain (13). The CSF system is important for the homeostasis of the CNS. It facilitates communication between the central nervous, the vascular, and immune systems. Soluble proteins and other macroscopic metabolic waste products are in part cleared from CSF by a recently discovered glymphatic system (14). An affected glymphatic system has been associated with cognitive decline in the elderly (15) and there are indications of a causal link between the progression of neurodegenerative diseases and diminished solute clearance (16).

The two aims of the present study were to evaluate if a targeted profiling of the CSF proteome, primarily including inflammatory and structural brain antigens, would (a) differ between preterm and term infants, and (b) predict neurodevelopmental outcome in preterm infants at 18–24 months corrected age. Our hypothesis was that protein concentrations would be different between the infants born preterm and the infants born at term age. Furthermore, that preterm infants with an unfavorable outcome might present a specific proteomic signature compared with the infants with favorable outcome. This was investigated through quantifications of CSF inflammatory mediators as well as structural and functional proteins related to brain development.

## Materials and methods

### Study participants and data acquisition

Twenty-seven preterm infants and 10 term-born infants were prospectively enrolled from the neonatal intensive care unit at Karolinska Hospital in Stockholm, Sweden, between January 2002 and January 2005, with informed parental consent. The median [inter quartile range (IQR)] gestational age was 27 w + 4 d (25 + 2 to 30 + 6) and 40 w + 6 d (38 + 0 to 41 + 6) for the preterm and term infants, respectively. All enrolled infants underwent clinically indicated lumbar puncture for suspected infection, and all received antibiotic treatment. Measurement of clinically established markers of infection in blood was performed in correlation with the lumbar puncture. Bacterial and viral cultures were performed in blood and CSF as per clinical routine. Twelve out of 49 eligible infants were excluded for one of the following reasons: intraventricular hemorrhage grade 2 or more according to Papile et al. (17), signs of periventricular leukomalacia on cranial ultrasound around the time of CSF collection, perinatal asphyxia or any other findings that indicated brain encephalopathy.

A neurodevelopmental follow-up was carried out in accordance with the Karolinska neonatal follow up program for all preterm infants. It was performed by a senior neonatologist and a physiotherapist until 18–24 months corrected postnatal age. We chose this interval to minimize loss of follow-up. Infants who presented abnormal neurodevelopment at that time were assessed with Bayley Scales of Infant and Toddler Development, Second Edition (BSID-II) (18).

All the term infants were examined by a senior neonatologist before discharge. They were not followed further in the neonatal follow-up program but had their check-ups within the regular childcare center, from where the information of the 18–24 month follow up was retrieved. Adverse neurodevelopmental outcome was defined as: neuromotor delay, cerebral palsy, seizure disorder, mental developmental index < 85, deafness or blindness, for preterm and term infants.

### Collection of cerebrospinal fluid

The median (IQR) age at CSF collection in preterm infants was 11 postnatal days (3.5–19.5 days) while at median 2 postnatal days (1–2.5 days) in term infants. Lumbar punctures were performed for clinical routine laboratory analysis in all patients and an additional 0.2–0.8 ml collected for research purposes. Samples were spun at 3,000 revolutions per minute (rpm) for 10 min and then the supernatants stored at −80°C until analyzed. Before analysis, the samples were thawed, and processed as previously described (12).

### Cerebrospinal fluid analysis

For the protein profiling in CSF a targeted antibody suspension bead array was used. It included 220 antibodies targeting 178 unique proteins (Supplementary Table 1). For the analysis, highly to moderately brain enriched proteins that are involved in different brain functions as well as a selection of proteins involved in neuroinflammatory processes, as recently described by Lindblad et al. (19) were chosen from the Human Protein Atlas (Science for Life Laboratory, Stockholm, Sweden) (20). Two “sibling”-antibodies targeting different regions of the same protein were included for 43 of the proteins.

Antibodies for the creation of the suspension bead array were produced from protein fragments (PrESTs) produced in *E. coli* and then immunized into rabbits after purification (21). Activation of the color-coded magnetic beads (500 000 beads per identity, MagPlex Luminex Corp.) and immobilization of the antibodies was performed as previously described (22). Sodium hydrogen phosphate 0.1 M, sulfo-NHS (Nordic Biolabs) 0.5 mg and EDC (ProteoChem) 0.5 mg per antibody were used to activate the beads surface. Then an incubation of the beads for 20 min at room temperature was performed in order to immobilize the selected antibodies to the different bead identities. A total concentration of 17.5 ng/microL of each antibody was assigned a specific bead ID. This was followed by washing off unwanted antibody excess with 0.1% Tween-20 after which the beads were blocked overnight using Roche blocking reagent for ELISA (supplemented with 400 microL Tween20) and lastly combined to form a suspension array.

The samples were processed as previously described (12). An assay buffer [BSA (Sigma-Aldreick)] 37.5 mg/mL, PBS (Fisher Scientific) 0.05% and rIgG 15 mg/mL (Bathyl Laboratories Inc., Montgomery, Texas, United States) were used to dilute the samples 1:2 after which they were randomized into 96-well microtiter plates. A labeling of the samples was performed using 10 × molar excess of biotin (NHS-PEG4-Biotin, Thermo Scientific) over protein amount, as previously described (23). Tris-HCl, 250 × over biotin amount was used to stop the reaction and an assay buffer (PVXCas) used for another 1:8 dilution. The samples were then put in a water bath for 30 min heat treatment at 56°C and after cooling incubated with the bed array overnight at room temperature in combinations of 45 microL of sample with 5 microL of bead array solution. Using paraformaldehyde (0.4% in PBS) the captured proteins were cross-linked to the antibodies. After a second wash a detection reagent (streptavidin conjugated R-phycoerythrin, 1:750 diluted in PBST, Invitrogen) was added. Finally, FlexMap3D instrument (Luminex Corp., Austin, Texas) was utilized to analyze the interacting proteins and report the relative protein abundances for each bead identity and sample as median fluorescent intensities.

### Statistics

Median and IQR were used to demonstrate the clinical and laboratory data. The results of the protein array analysis were presented as raw median fluorescent intensity. No normalization was performed, due to the low numbers of samples available. For simplification, the median fluorescent intensity differences of CSF protein abundances were visualized on a Volcano plot after log-2 transformation of the data into fold changes. The differences of the CSF protein levels between preterm and term infants as well as the preterm outcome groups, were analyzed using Mann-Whitney *U*-test, chosen because of small sample size and non-normally distributed data. The differences were considered statistically significant if *p*-value was < 0.05. The results of proteins representing the largest median fluorescent intensity differences between preterm and term infants and between preterm outcome groups are presented on scatter plots. *p* < 0.001, to be included in figures.

## Results

### Patient characteristics

The patient characteristics are summarized in Table 1. Gestational age, birth weight, postnatal age at lumbar puncture and number of verified infections differed between the preterm and term infant groups but not the C-reactive protein levels. Eleven of the preterm infants had adverse neurological outcome at 18–24 months follow-up assessment, but none of the term infants. While complete outcome data was missing for *n* = 3 preterm infants, national records showed that they were all alive at 24 months. No significant differences were found in gestational age, postnatal age at lumbar puncture or C-reactive protein level in the preterm group divided by outcome (Table 2). Sixteen out of 26 preterm infants with available culture results had culture verified blood stream and/or CSF infection, 8/13 (62%) with normal outcomes and 8/11 (72%) with unfavorable outcomes, respectively. All term infants had negative CSF- and blood cultures.

**TABLE 1:** Clinical characteristics of preterm and term infants.

	Term infants	Preterm infants
Number of patients (*n*)	10	27
Gestational age (weeks + days)	40 + 6 (38 + 0 to 41 + 5)	27 + 5 (25 + 3 to 30 + 6)
Birth weight (g)*	3,900 (3,627 to 4,943)	850 (707 to 1,117)
Age at lumbar puncture (days)*	2 (1 to 2.25)	11 (4 to 21)
C-reactive protein	92 (71 to 110)	75 (14 to 96)
Positive blood or CSF cultures (*n*)*	0/10	16/26**

Data are presented as median (IQR). *p < 0.001, ** n = 26 of the preterm infants had available data on blood and CSF cultures. CSF, cerebrospinal fluid.

**TABLE 2:** Neurodevelopmental outcome.

	Preterm infants with adverse outcome*	Preterm infants with normal outcome
	(*n* = 11)	(*n* = 13)
Gestational age (weeks + days)	26 + 3 (25 + 1 to 28 + 2)	27 + 4 (25 + 2 to 31 + 0)
Birth weight (g)	912 (707 to 1,005)	834 (659 to 1,454)
Age at lumbar puncture (days)	8 (2 to 15)	11 (5 to 28)
C-reactive protein	70 (7 to 90.5)	94 (30 to 124)
Positive blood or CSF cultures (*n*)	8	8

Data are presented as median (IQR). *Adverse outcome was defined as: cerebral palsy, seizure disorder, motor delay, mental developmental index < 85, deafness or blindness at 18–24 months assessment.

### Differences in protein profiles between preterm and term infants

**FIGURE 1:**
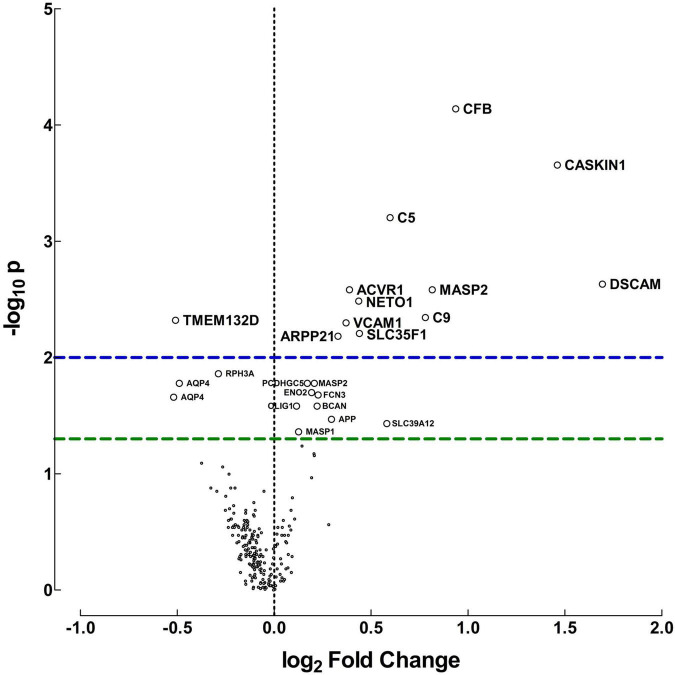
Protein differences between preterm and term infants. Volcano plot for visualization of the differences in relative protein abundances between preterm and term infants. The plot was created by transforming the median fluorescent intensity differences into log_2_ fold changes and plot it against −log_10_ *p*-values, calculated with Mann-Whitney *U*-test. Fold changes above zero indicate higher protein levels in CSF of preterm infants, but below zero indicates higher levels in term infants. Horizontal dashed lines indicate significance, green: *p* = 0.05, blue: *p* = 0.01.

A distinct CSF proteome reflecting ongoing neurodevelopmental and neuroinflammatory processes was observed in preterm infants, Figure 1. The relative protein abundances detected in CSF samples, represented as median fluorescent intensity differences are transformed into log_2_ fold changes for visualization.

Proteins related to the innate immune response and neurodevelopment exhibited the biggest difference in median fluorescent intensities and fold changes between preterm and term infants. These included complement factor B (CFB), complement 5 (C5), complement 9 (C9) and mannose-binding protein-associated serine protease 2 (MASP). Moreover, proteins involved in neurodevelopmental regulation and synaptic plasticity were also present in higher concentrations in preterm vs. term infants. These included calcium/calmodulin-dependent serine protein kinase (CASK)-interacting protein 1 (CASKIN1), cyclic AMP-regulated phosphoprotein 21 (ARPP21), Neurophilin and tolloid like proten 1 (NETO1) and Down syndrome cell adhesion molecule (DSCAM). Only a handful of proteins were found increased in term infants compared with preterm.

The protein differences between preterm and term infants were consistent regardless of culture results. When protein levels were compared in preterm infants with positive cultures and preterm infants with negative cultures, no differences were found (Supplementary Table 2). As the term infants all had favorable outcomes, an additional comparison was made between the protein levels of term and preterm infants with normal outcomes. Similar protein concentrations were found as described above, but for most proteins the differences were larger in this subset. Significant differences were found for 35 proteins, *p*-value < 0.05. The proteins that exhibited the highest median fluorescent intensity data differences and lowest *p*-values, are displayed in Table 3. All protein differences are shown in Supplementary Table 3.

**TABLE 3:** Protein alterations between term and preterm infants with normal outcome.

			Preterm		Term				
Analyte	Description	Function	Median	IQR	Median	IQR	ΔMFI	log_2_ Fold Change	*p* *
CFB	Complement factor B	Neuroinflammation, regulation of immune reaction	3,448	3,222–4,678	1,802	1,643–2,209	1,646	**0.94**	7.21E-05
C5	Complement 5	Neurodevelopment and neuroinflammation	1,143	961–1,249	686	588–751	438	**0.74**	1.98E-04
CASKIN1	CASK-interacting protein 1	Synaptic plasticity	3,753	2,294–5,863	1,022	790–1,521	2,731	**1.88**	1.98E-04
VCAM1	Vascular cell adhesion molecule 1	Neuroinflammation and early neural proliferation	1,672	1,478–2,002	1,159	1,105–1,411	513	**0.53**	5.15E-04
ARPP21	Cyclic AMP-regulated phosphoprotein 21	Synaptic plasticity	1,109	970–1,203	799	701–905	310	**0.47**	7.25E-04
MASP2	Mannose-binding protein-associated serine protease 2	Neural migration and neuroinflammation	1,730	1,353–3,899	982	824–1,168	748	**0.82**	8.11E-04
ACVR1	Activin receptor type-1	Neurotrophic and bone morphogenic properties	1,165	1,075–1,431	873	770–953	292	**0.42**	1.26E-03
BCAN	Brevican	Brain developmental and plasticity, maintenance of neural circuitry	1,863	1,581–1,977	1,424	1,164–1,636	439	**0.39**	1.93E-03
NETO1	Neurophilin and tolloid like proten 1	Synaptic plasticity	993	829–1,477	712	658–865	281	**0.48**	1.93E-03
DSCAM	Down syndrome cell adhesion molecule	Brain development and neurotrophic properties	8,685	4,537–11,743	2,158	938–4,267	6,527	**2.01**	1.93E-03
NSE	Neuron specific enolase	Neurotrophic and neuroprotection properties	1,222	1,035–1,502	968	892–1,085	254	**0.34**	2.37E-03
APP	Amyloid beta precursor protein	Synaptogenesis and synaptic plasticity	4,460	3,899–4,970	3,264	2,834–3,942	1,196	**0.45**	4.33E-03

Proteins in cerebrospinal fluid that exhibited differences in median fluorescent intensity levels at the threshold of p < 0.005 between term infants and preterm infants with outcome, established by Mann- Whitney U-test. ΔMFI, median fluorescent intensity differences; IQR, inter quartile range. *p ≤ 0.005. Bold used to highlight the “fold changes”.

**FIGURE 2:**
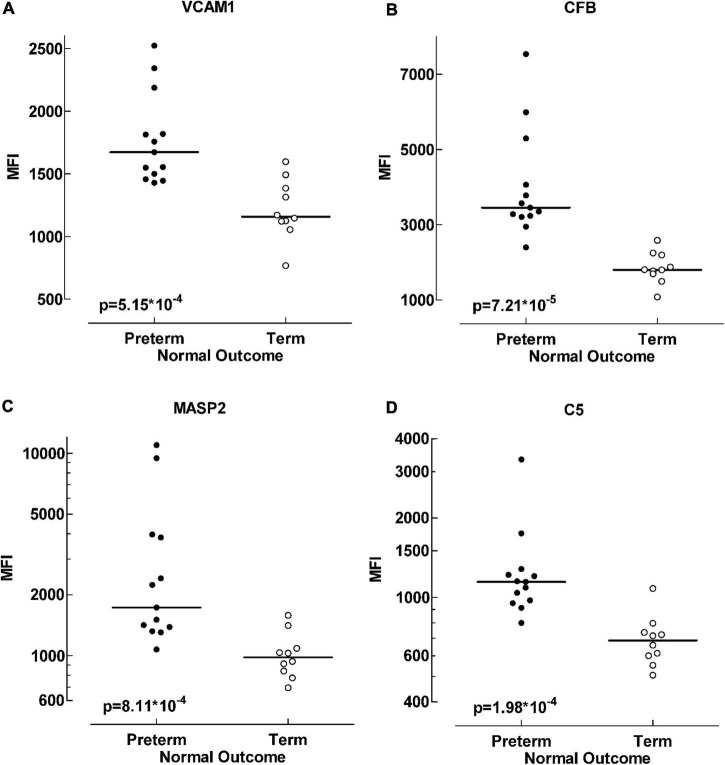
Protein differences between term and preterm infants with normal outcome. Scatter plot visualizing alterations in protein levels between preterm and term infant groups. Four of the proteins exhibiting the largest data differences; VCAM1 (vascular cell adhesion molecule 1) **(A)**, CFB (complement factor B) **(B)**, MASP2 (mannose-binding protein associated serine protease 2) **(C)** and C5 (complement factor 5) **(D)**. Data analyzed with Mann-Whitney *U*-test, *p* < 0.001.

The four inflammatory proteins that differed the most between these patient groups are displayed in more detail in Figure 2.

### Proteins predicting outcome in preterm infants

**FIGURE 3:**
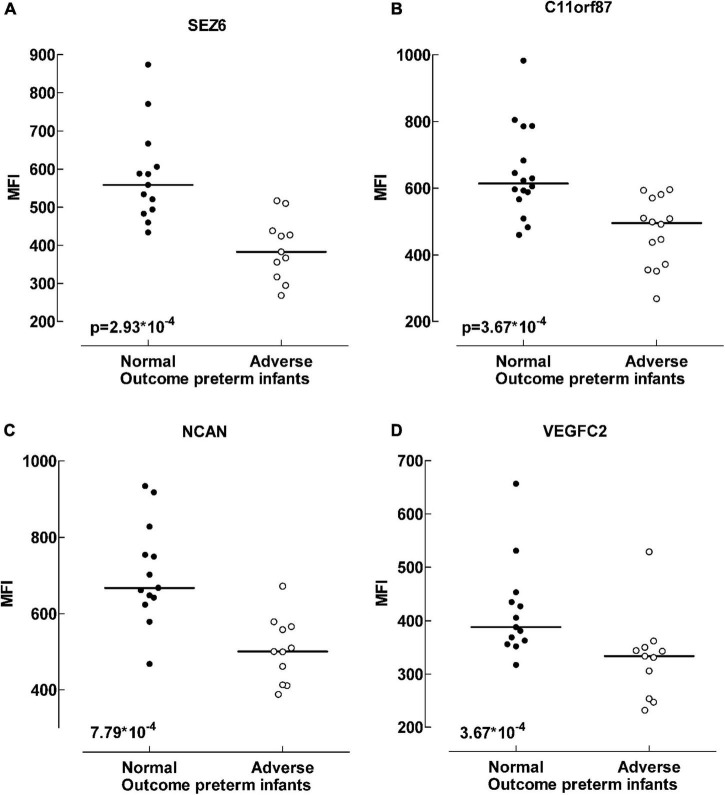
Protein differences between outcome groups. Scatter plot visualizing differences in median fluorescent intensity levels between outcome groups of preterm infants. Four of the proteins that exhibited the largest differences; Sez 6 (seizure protein 6) **(A)**, C11orf87 (neuronal integral membrane protein 1) **(B)**, NCAN (neurocan core protein) **(C)** and VEGFC (vascular endothelial growth factor C) **(D)**. Adverse outcome was defined as neuromotor delay, cerebral palsy, seizure disorder, mental developmental index < 85, deafness or blindness at 18–24 months assessment. Data analyzed with Mann-Whitney *U*-test, *p* < 0.001.

A differential analysis was conducted to compare the protein levels in preterm infants with favorable and unfavorable outcomes. Several proteins were negatively correlated with unfavorable outcome in preterm infants (Supplementary Table 4). In Table 4 the proteins that differed the most between the outcome groups are highlighted (*p* < 0.001). Most are proteins essential for brain function and development. These proteins were consistently found at lower levels in preterm infants with adverse outcome, regardless of culture results (data not shown). In Figure 3, four of the proteins with the biggest alterations in median fluorescent intensity between the outcome groups are displayed.

**TABLE 4:** Protein alterations predicting outcome in preterm infants.

			Normal outcome		Adverse outcome				
Analyte	Description	Function	Median	IQR	Median	IQR	ΔMFI	log_2_ Fold Change	p*
SEZ6	Seizure protein 6	Neural growth and differentiation	558	489–637	383	317–438	175	**0.5**	2.93E-04
IL1A	Interleukin 1 alpha	Regulates immune responses	756	719–869	658	566–677	98	**0.2**	2.93E-04
C11orf87	Neural integral membrane protein 1	Ion transport in brain cells	630	595–786	499	372–581	131	**0.3**	3.67E-04
VEGFC	Vascular endothelial growth factor C	Promotes angiogenesis	524	490–566	414	353–443	110	**0.3**	3.67E-04
NKAIN2	Na^+^/K^+^ transporting ATPase interacting protein 2	Nervous system development	727	708–865	652	550–670	75	**0.15**	4.09E-04
HPCA	Hippocalcin	Neurogenesis and astrocytic differentiation regulation	484	442–539	415	337–427	69	**0.2**	6.30E-04
TGFB2	Transforming growth factor beta 2	Brain development and neuroprotection	738	711–835	646	542–677	92	**0.2**	7.01E-04
TBR1	Neuron-specific T-box transcription factor	Cortical development	806	780–914	730	642–783	76	**0.14**	7.01E-04
NCAN	Neurocan core protein	Neuronal migration and plasticity	667	633–791	501	413–566	166.5	**0.4**	7.79E-04
GPM6B	Glycoprotein M6B	Neuronal differentiation and myelination	693	641–788	617	540–640	76	**0.16**	7.79E-04
DIRAS2	GTP-binding RAS-like protein 2	Synaptic function in hippocampus and cerebral cortex	523	488–573	452	372–483	**72**	**0.2**	8.64E-04

Cerebrospinal fluid proteins exhibiting significant median fluorescent intensity differences between preterm infants with adverse outcome and preterm infants with normal outcome, at threshold of p < 0.001, established by Mann-Whitney U test. Adverse outcome was defined as neuromotor delay, cerebral palsy, seizure disorder, mental developmental index < 85, deafness or blindness at 18–24 months assessment. ΔMFI, median fluorescent intensity differences; IQR, inter quartile range. *p ≤ 0.001. Bold used to highlight the “fold changes”.

## Discussion

We show a combination of inflammatory and neurodevelopmentally related CSF proteomic signatures in preterm infants in the neonatal period. Employing the sensitivity of a targeted antibody microarray assay, we identified increased concentrations of 35 proteins in preterm infants compared with term infants. Notably, adverse neurodevelopmental outcome in preterm infants correlated with lower protein levels.

### Neuroinflammation and brain development

An upregulation of proteins associated with brain development, synaptic plasticity and neuroinflammation was observed in the preterm group compared with term infants.

Proteins that differed the most were proteins associated with the complement system, including CFB, C5, and MASP2, and the inflammation regulating protein vascular cell adhesion molecule 1 (VCAM). Increased levels of inflammatory proteins in CSF, including the proteins of the complement cascade, have previously been associated with preterm birth (11, 21). Components of the complement system have been shown to be produced locally in the brain (24, 25). They contribute to tissue damage in the injured or diseased brain (26). There is also evidence of the involvement of the complement system in brain development and altered neuroplasticity following postnatal events (27). In a mouse model of inflammation induced preterm birth, C5 was associated with decreased expression of neural markers and cortical abnormalities (28). However, opposing roles have been observed, and C5 is also associated with neuroprotection and normal neurodevelopment (29, 30). Proliferation of neural progenitor cells was observed in mouse embryos treated with C5aR agonist (31). VCAM also has an important role in brain development. It is essential for the proliferation of neural stem cells in early development and the lack of VCAM leads to dysmaturation of neural progenitor cells (32).

Immunoregulatory proteins can have both detrimental and beneficial effects. Most of the proteins that were present in higher concentrations in preterm infants with normal outcome vs. term controls seem to be involved in normal brain development (9). DSCAM is one of these proteins and was found higher in preterm infants, in comparison with term infants. It is involved in neurite guidance and synapse formation (33) as well as in the innate immune response. Increased concentrations of several other neurodevelopmentally related proteins were observed in the preterm population compared with term infants. This likely reflect the rapid neurodevelopment and growth during the early developmental stages of the preterm brain, which might explain in part the vulnerability of the preterm brain to a diversity of external stimuli.

The data differences between preterm and term infants were even larger when only the preterm and term infants with normal outcome were compared. One of these was activin receptor type-1 (ACVR1), which is a transmembrane kinase receptor for members of the transforming growth factor-β (TGFB) family with neurotrophic and neuroprotective properties (34, 35). Moreover, several proteins primarily localized in synapsis and involved in cell interaction and synaptogenesis were found higher in the preterm population. These included CASKIN1, ARPP21 and amyloid beta precursor (APP), important post-transcriptional regulators of dendritic growth as well as synaptic formation and plasticity (36, 37). Recently, experimental studies suggested a correlation between the serine protein kinase CASKIN1 and stress response and memory formation (37, 38). Moreover, human mutations in CASKIN1 is present in neurodevelopment disorders and correlated to presynaptic dysfunction (39). CASKIN1 alterations have in preclinical rodent models been seen to be responsible for the presentation of autism spectrum disorder (ASD) behavioral phenotypes (40).

Neither blood or CSF culture results nor C-reactive protein levels did influence the observed protein differences. However, despite these results systemic infections in infants cannot be fully excluded (41, 42). Preterm infants might have increased susceptibility to severe disease partly as a result of naïve immune status and compromised responsiveness of immune system (43). Recently, distinct, and unified blood protein profiles were observed in preterm infants directly at birth while different trends of the infants’ blood protein profiles were observed shortly after, depending on diverse postnatal events (44). Differences in plasma protein profiles in preterm infants have also been correlated with changes in gestational age (45).

Few proteins were found in higher concentrations in the term group compared with the preterm infants. Of these, transmembrane protein 132D differed the most. This is a protein found in mature oligodendrocytes and therefore not yet fully expressed in the preterm infant’s brain. Also, aquaporin-4 (AQP4) protein was higher in term than in preterm infants. AQP4 is a CSN water channel involved in part in the glymphatic CSF clearance system and its dysfunction has been correlated with cognitive decline in the elderly (14, 15). Whether lower levels of AQP4 in preterm infants indicate a less mature glymphatic system needs further evaluation. For the investigation of the glymphatic function in different conditions, simultaneous analysis of protein concentrations in peripheral blood and CSF would be preferable.

### Markers of adverse neurodevelopmental outcome

It has been suggested that neuroinflammation may promote neurodevelopmental impairment by altering the expression of developmental genes (46, 47). However, the exact molecular pathways and mechanisms leading to adverse neurodevelopmental outcome is not fully understood.

We have recently revealed how the proteomic profile after perinatal asphyxia can predict death and long-term disability in term infants. In that report we revealed several novel protein biomarkers, that were elevated in term infants with adverse outcome (48). In contrast to term infants, here lower CSF levels of several mediators of neurodevelopment predicted outcome of preterm infants. Recently, Zhong et al., observed trends of both up- and downregulations of protein profiles in 14 preterm infants, depending on exposure to different postnatal events (44). In that study correlation of the plasma protein profiles to neurodevelopmental outcomes was not performed. However, decreased protein levels in serum of those same 14 infants were associated with severe retinopathy of prematurity (49). Retinopathy of prematurity is a disease that is associated with the lowest gestational ages and its severity increases with increasing prematurity. Both up- and downregulation of several mediators of neurodevelopment was shown in a study on preterm infants with post hemorrhagic ventricular dilatation, compared with healthy term infants (50). In that study several of the observed proteins were found in lower amounts after initiation of medical treatment. No correlation with clinical outcome was performed but post hemorrhagic ventricular dilatation is a condition that carries a high risk of neurodevelopmental adversities. In a recent study downregulation of neurotrophic proteins was correlated with lower brain volumes and later impaired cognitive development in preterm infants (46). Thus, our data on CSF protein signatures, is in line with these studies. Moreover, lower levels of neurodevelopmental proteins seem to be associated with cognitive decline in the elderly (51, 52). Notably, in these studies the proteomic results in the CSF are more robust than in peripheral blood. This supports the current approach of using CSF instead of blood, though we would want to further research to validate our findings in additional biofluid compartments.

Particularly, the present study highlights 11 proteins (Table 4), that strongly associate with long term outcome of preterm infants. The proteins that differed the most between outcome groups were regulatory proteins of brain development. These included vascular endothelial growth factor C (VEGFC), that promotes and regulates angiogenesis in brain both during development and repair. It has also been shown to have trophical effects on neural progenitor cells (53). Serum VEGF concentrations of preterm infants are dynamic the first week of life (54). Recently the administration of VEGFC was shown to improve neurological repair following traumatic brain injury in a rat model through modulating microglial activation (52). Thus, low levels in preterm infants are likely associated with inadequate angiogenesis and a hampered neurogenesis.

Neurocan core protein (NCAN) and TGFB levels also correlated with outcome. NCAN is a component of the extracellular matrix in the brain and has an important role in neuronal migration and plasticity, especially the development of the cortex (55). TGFB is a multifunctional cytokine that enhances the expression of NCAN (56). The lack of NCAN has been associated with cognitive decline and diminished brain volumes in the elderly (51). These findings are in contrast with findings of experimental study where upregulation of these proteins was observed in traumatically injured brain tissue and correlated with delayed axonal repair (56). These differences could be due to different underlying causes. The neurological adversities of preterm infants, like in the elderly, are in large part due to disturbances in brain development, while the sequelae of traumatic brain injury stem from lesions following an insult, and the formation of scar tissue. Another mediator of cortical development and essential for cognitive development is neuron-specific T-box transcription factor (TBR1) (57). This was one of the protein that was found at lower levels in preterm infants with adverse outcome compared with normal. Mutations in Tbr1 gene caused synaptic and neuronal dysfunctions that led to ASD like behavior (58). Preterm infants are at significantly higher risk at developing ASD than children born at term age (59, 60).

Seizure protein 6 (Sez6) and Na^+^/K^+^ transport ATPase interacting protein 2 (NKAIN2) are essential proteins in nervous systems’ health and development. Lower levels of these proteins were correlated with adverse outcome of preterm infants in the present study. NKAIN2 is associated with myelination. Chromosomal deletion of the region responsible for this protein has been correlated with both cerebral atrophy and abnormal white matter development (61, 62). The lack of Sez6 has been linked with impairment in motor functions, memory, and cognition in experimental studies (63). Another protein that was lower in the adverse outcome group was chromosome 11 open reading frame 87 (c11orf87) protein, also known as neuronal integral membrane protein 1. It is primarily expressed in brain tissue and suppressed levels have been correlated to schizophrenia related pathways (64). Notably, dysfunction of synaptic transmission, synaptic plasticity and behavior pathways in brain has been implicated in schizophrenia (65). Preterm birth, especially before week 32, is associated with a 3–4-fold increased risk of developing neuropsychiatric disorders, including schizophrenia (66). To the best of our knowledge, we are the first to report relatively lower levels of c11orf87 protein in the preterm CSF. Future work could delineate whether its expression is transcriptionally or epigenetically regulated in the preterm brain.

### Clinical implication

Preterm birth is associated with complex brain abnormalities resulting from immaturity. Early prediction and better understanding the mechanisms for neurodevelopmental outcome in this high-risk population is crucial. It would allow more individualized support to minimize neurological sequalae. Protein profile analysis is a methodology that provides insight into the biological processes that the proteins participate in. Thus, revealing changes in these processes, that might lead to aberrant neurodevelopment.

Although many questions are unresolved about the exact pathological process of developmental brain injury, neuroinflammation is an important contributor. Therefore, inflammatory proteins in CSF are of clinical importance. Furthermore, many questions are yet to be answered about the role of the different neurodevelopmentally related proteins. The present study contributes to the knowledge of the physiological changes that occur in the developing brain as measured by markers in CSF. Thus, it may aid in the identification of infants at risk for adverse neurodevelopmental outcome. Therapeutic options for preterm brain injury are currently limited. The present study indicates that preterm infants with adverse outcome have lower CSF levels of several of the proteins critical for brain development, neuro- and synaptogenesis. This is in line with experimental studies in stroke models, where VEGFC treatments diminished brain injury and enhanced recovery (67). Notably, in other pathological conditions of premature birth, the blocking of proteins may enhance recovery, as has been established with anti-VEGF treatment in retinopathy of prematurity (68).

Parenteral nutrition initiated earlier, with higher amounts of protein and fat improve the growth of extremely preterm infants including their head circumference (69, 70). We speculate that part of the mechanism is to provide the developing brain with crucial proteins enabling adequate brain development and better overall outcome. To provide enough macronutrient intake, including protein substrates, is a modifiable factor that could reduce the risk of adverse neurodevelopmental outcomes for these fragile infants.

### Strengths and Limitations

The strengths of this study include the unique biobank of CSF from preterm and term infants, collected under clinical routine care. We also have performed an in-depth broad analysis of protein profiles in CSF, using a technique that simultaneously evaluates the levels of 178 proteins in small samples. This enabled us to provide insight into pathophysiological changes that may lead to adverse neurodevelopment in preterm infants.

We acknowledge some limitations of the present study. All infants included in the study had clinical and laboratory signs of infection and a substantial part of the preterm group had positive cultures in blood and/or CSF (16/26). However, in the term group, all infants showed negative culture results. The age of the infants at the time of CSF collection varied, with the term group being significantly younger than the preterm group. The protein selection is inevitably biased as it was chosen based on previous studies. Many of the analyzed proteins have correlations with infection and brain development. Also, we included proteins that have been studied in brain ischemia and other forms of brain trauma, for which more research is available in older children and adults. Another limitation is the assessment of neurodevelopmental outcome that was done in a non-standardized way and only until 18–24 months corrected age. This is due to the fact, that follow-up was not a part of the study protocol, but was conducted according to current clinical routine. Therefore, it differed depending on degree of prematurity and on clinical findings. While we have relatively large cohort in context of what has previously been published (11, 71), it is possible that the study is underpowered to reveal a significant difference for a subset of our analyzed proteins. Thus, additional, larger studies are warranted to reproduce our findings and establish possible type-2 errors in the current study.

## Conclusion

Numerous protein abundances in preterm infants in comparison with term infants, were demonstrated, the upregulation of proteins related to neuroinflammation being the most prominent. Preterm infants with unfavorable outcome shared a specific proteomic profile in CSF, with generally lower levels of functional and structural brain related proteins, in comparison with preterm infants with favorable outcome. In the present study the CSF proteomic signatures of preterm infants observed may reflect developmental changes in response to external or internal events and have value in predicting long-term outcome. The study contributes to the understanding of the neurodevelopmental processes in the preterm brain. It renders novel data regarding essential proteins for optimal neurodevelopmental outcome and suggests a vulnerability if these proteins are decreased. Thus, this opens up possibilities of tentative treatment options for preterm infants, including supplementary proteins or protein substrates.

## Data Availability Statement

The original contributions presented in this study are included in the article/Supplementary Material, further inquiries can be directed to the corresponding author/s.

## Ethics statement

The regional Ethical Review Board at the Karoliska Institute and Stockholm County (Dnr 98-246, 2003-174, 2011/1891-31) approved this study. It was carried out in accordance with European Community guidelines and the Declaration of Helsinki. Written informed consent to participate in this study was provided by the participants’ legal guardian/next of kin.

## Author contributions

KL, VS, ET, and EH conceptualized and designed the study. KL, EH, and VS enrolled the patients and collected the samples. KL, EH, KJ, and VS collected and interpreted clinical data. PN and ET were responsible for sample analysis. KL, EH, ET, and KJ analyzed the data. KL, PL, and SE did statistical analysis. KL, ET, EH, KJ, ÁH, and VS wrote the manuscript. All authors read and approved the final manuscript.

## Conflict of Interest

The authors declare that the research was conducted in the absence of any commercial or financial relationships that could be construed as a potential conflict of interest.

## Publisher’s Note

All claims expressed in this article are solely those of the authors and do not necessarily represent those of their affiliated organizations, or those of the publisher, the editors and the reviewers. Any product that may be evaluated in this article, or claim that may be made by its manufacturer, is not guaranteed or endorsed by the publisher.

## Abbreviations

ACVR1: activin receptor type-1
APP: amyloid beta precursor protein
AQP4: aquaporin-4
ARPP21: cyclic AMP-regulated phosphoprotein 21
c11orf87: chromosome 11 open reading frame 87
ASD: autism spectrum disorder
CASKIN1: calcium/calmodulin-dependent serine protein kinase (CASK)-interacting protein-1
C5: complement 5
C5Ar: C5a receptor
C9: complement 9
CFB: complement factor B
CNS: central nervous system
CSF: cerebrospinal fluid
DSCAM: Down syndrome cell adhesion molecule
IQR: inter quartile range
MASP2: mannose-binding protein-associated serine protease 2
NCAN: Neurocan core protein
NETO1: Neurophilin and tolloid like proten 1
NKAIN2: Na^+^/K^+^ transport ATPase interacting protein 2
SEZ6: Seizure protein 6
TGFB: transforming growth factor-β family
VCAM1: vascular cell adhesion molecule1
VEGFC: vascular endothelial growth factor C.
