# Supplementary material for: The cerebrospinal fluid proteome of preterm infants predicts neurodevelopmental outcome
Source: Front Pediatr. 2022 Jul 19;10:921444. doi: 10.3389/fped.2022.921444 (PMC9343678; doi:10.3389/fped.2022.921444)
Supplement: Supplementary file 1 [file Data_Sheet_1.PDF]

Supplementary Table 1

List of all proteins and antibody IDs

**Analyte**	**Description**	**UniProt ID**	**HPA* Antibody ID**
ACVR1	Activin A receptor type 1	Q04771	HPA007505
ACVR1	Activin A receptor type 1	Q04771	HPA008014
ACVR1C	Activin A receptor type 1C	Q8NER5	HPA007982
AK5	Adenylate kinase 5	Q9Y6K8	HPA019128
AK5	Adenylate kinase 5	Q9Y6K8	HPA057255
ALDOC	Aldolase fructose-bisphosphate C	P09972	HPA003282
ALDOC	Aldolase fructose-bisphosphate C	P09972	HPA067442
AMER2	APC membrane recruitment protein 2	Q8N7J2	HPA039458
APC2	APC2 WNT signaing pathway regulator	O95996	HPA078002
APP	Amyloid beta precursor protein	P05067	HPA001462
APP	Amyloid beta precursor protein	P05067	HPA031303
AQP4	Aquaporin 4	P55087	HPA014784
AQP4	Aquaporin 4	P55087	HPA014944
ARPP21	CAMP regulated phosphoprotein 21	Q9UBL0	HPA017303
ATP6V1G2	ATPase H+ transporting V1 subunit G2	O95670	HPA068667
AVP	Arginine vasopressin	P01185	HPA071892
BAALC	BAALC MAP3K1 and KLF4 binding	Q8WXS3	HPA027132
BAALC	BAALC MAP3K1 and KLF4 binding	Q8WXS3	HPA077738
BCAN	Brevican	Q96GW7	HPA007865
BTBD17	BTB domain containing 17	A6NE02	HPA025022
C11orf87	Chromosome 11 open reading frame 87	Q6NUJ2	HPA034656
C1orf61	Chromosome 1 open reading frame 61	Q13536	HPA076077
C1QA	Complement C1q A chain	P02745	HPA002350
C1QB	Complement C1q B chain	P02746	HPA052116
C1QL2	Complement C1q like 2	Q7Z5L3	HPA057934
C2orf80	Chromosome 2 open reading frame 80	Q0P641	HPA078078
C5	Complement C5	P01031	HPA075945
C8orf46	Chromosome 8 open reading frame 46	Q8TAG6	HPA075134
C9	Complement C9	P02748	HPA029577
C9	Complement C9	P02748	HPA070709
CACNG3	Calcium voltage-gated channel auxiliary subunit gamma 3	O60359	HPA059010
CACNG8	Calcium voltage-gated channel auxiliary subunit gamma 8	Q8WXS5	HPA041351
CAMK2G	Calcium/calmodulin dependent protein kinase II gamma	Q13555	HPA051783
CAMK2G	Calcium/calmodulin dependent protein kinase II gamma	Q13555	HPA051785
CASKIN1	CASK interacting protein 1	Q8WXD9	HPA055990
CASKIN1	CASK interacting protein 1	Q8WXD9	HPA076882
CCL18	C-C motif chemokine ligand 18	P55774	HPA047485
CFB	Complement factor B	P00751	HPA001817
CFI	Complement factor I	P05156	HPA024061
CFI	Complement factor I	P05156	HPA001143

CHRNB2	Cholinergic receptor nicotinic beta 2 subunit	P17787	HPA062865
CLEC7A	C-type lectin domain containing 7A	Q9BXN2	HPA050229
CNTNAP4	Contactin associated protein like 4	Q9C0A0	HPA031859
CNTNAP4	Contactin associated protein like 4	Q9C0A0	HPA057342
CREG2	Cellular repressor of E1A stimulated genes 2	Q8IUH2	HPA057596
CSPG5	Chondroitin sulfate proteoglycan 5	O95196	HPA071779
CSPG5	Chondroitin sulfate proteoglycan 5	O95196	HPA076601
CXCL1	C-X-C motif chemokine ligand 1	P09341	HPA067614
DIRAS2	DIRAS family GTPase 2	Q96HU8	HPA043758
DLL3	Delta like canonical Notch ligand 3	Q9NYJ7	HPA060025
DSCAM	DS cell adhesion molecule	O60469	HPA019324
DSCAM	DS cell adhesion molecule	O60469	HPA074915
ELAVL3	ELAV like RNA binding protein 3	Q14576	HPA070436
ERC2	ELKS/RAB6-interacting/CAST family member 2	O15083	HPA073739
ERMN	Ermin	Q8TAM6	HPA038295
ERMN	Ermin	Q8TAM6	HPA038296
FABP7	Fatty acid binding protein 7	O15540	HPA061703
FAM181B	Family with sequence similarity 181 member B	A6NEQ2	HPA066861
FAM181B	Family with sequence similarity 181 member B	A6NEQ2	HPA075523
FCN1	Ficolin 1	O00602	HPA000685
FCN1	Ficolin 1	O00602	HPA001295
FCN2	Ficolin 2	Q15485	HPA076099
FCN3	Ficolin 3	O75636	HPA071173
FEZF2	FEZ family zinc finger 2	Q8TBJ5	HPA068604
FRMPD4	FERM and PDZ domain containing 4	Q14CM0	HPA075921
GABRA1	Gamma-aminobutyric acid type A receptor alpha 1 subunit	P14867	HPA055746
GABRA5	Gamma-aminobutyric acid type A receptor alpha 5 subunit	P31644	HPA059644
GABRB2	Gamma-aminobutyric acid type A receptor beta2 subunit	P47870	HPA067632
GABRG1	Gamma-aminobutyric acid type A receptor gamma1 subunit	Q8N1C3	HPA035622
GALNT17	Polypeptide N-acetylgalactosaminyltransferase 17	Q6IS24	HPA013624
GAP43	Growth associated protein 43	P17677	HPA013392
GDAP1L1	Ganglioside induced differentiation associated protein 1 like 1	Q96MZ0	HPA063265
GFAP	Glial fibrillary acidic protein	P14136	HPA056030
GFAP	Glial fibrillary acidic protein	P14136	HPA063513
GPM6A	Glycoprotein M6A	P51674	HPA017338
GPM6B	Glycoprotein M6B	Q13491	HPA002913
GPM6B	Glycoprotein M6B	Q13491	HPA077843
GPR26	G protein-coupled receptor 26	Q8NDV2	HPA062736
GPR37L1	G protein-coupled receptor 37 like 1	O60883	HPA052631
GPR37L1	G protein-coupled receptor 37 like 1	O60883	HPA064454
GRIA2	Glutamate ionotropic receptor AMPA type subunit 2	P42262	HPA008441
GRIA2	Glutamate ionotropic receptor AMPA type subunit 2	P42262	HPA070769
GRIN1	Glutamate ionotropic receptor NMDA type subunit 1	Q05586	HPA067773
GRIN2A	Glutamate ionotropic receptor NMDA type subunit 2A	Q12879	HPA004693
GRIN2A	Glutamate ionotropic receptor NMDA type subunit 2A	Q12879	HPA045139
GRM1	Glutamate metabotropic receptor 1	Q13255	HPA015701

GRM3	Glutamate metabotropic receptor 3	Q14832	HPA053434
HAPLN2	Hyaluronan and proteoglycan link protein 2	Q9GZV7	HPA045765
HIF1A	Hypoxia inducible factor 1 alpha subunit	Q16665	HPA001275
HIF3A	Hypoxia inducible factor 3 alpha subunit	Q9Y2N7	HPA041141
HIF3A	Hypoxia inducible factor 3 alpha subunit	Q9Y2N7	HPA074609
HPCA	Hippocalcin	P84074	HPA043245
HRH3	Histamine receptor H3	Q9Y5N1	HPA072396
HSPA4	Heat shock protein family A (Hsp70) member 4	P34932	HPA010023
HTR2A	5-hydroxytryptamine receptor 2A	P28223	HPA014011
HTR2C	5-hydroxytryptamine receptor 2C	P28335	HPA052903
IFNG	Interferon gamma	P01579	HPA053530
IL10	Interleukin 10	P22301	HPA071391
IL12A	Interleukin 12A	P29459	HPA001886
IL1A	Interleukin 1 alpha	P01583	HPA075911
IL1B	Interleukin 1 beta	P01584	HPA064606
IL1B	Interleukin 1 beta	P01584	HPA068737
IL4	Interleukin 4	P05112	HPA042270
IL4	Interleukin 4	P05112	HPA070010
IL6	Interleukin 6	P05231	HPA064428
JPH3	Junctophilin 3	Q8WXH2	HPA076304
KCNA1	Potassium voltage-gated channel subfamily A member 1	Q09470	HPA074471
KCNC1	Potassium voltage-gated channel subfamily C member 1	P48547	HPA041392
KCNC1	Potassium voltage-gated channel subfamily C member 1	P48547	HPA047634
KCNF1	Potassium voltage-gated channel modfier subfamily F member 1	Q9H3M0	HPA014738
KCNF1	Potassium voltage-gated channel modifier subfamily F member 1	Q9H3M0	HPA062278
KCNJ9	Potassium voltage-gated channel subfamily J member 9	Q92806	HPA070478
KCNQ3	Potassium voltage-gated channel subfamily Q member 3	O43525	HPA059375
KCNV1	Potassium voltage-gated channel modifier subfamily V member 1	Q6PIU1	HPA069362
KCNV1	Potassium voltage-gated channel modifier subfamily V member 1	Q6PIU1	HPA075000
KIF3C	Kinesin family member 3C	O14782	HPA075785
KIF5A	Kinesn family member 5A	Q12840	HPA004469
KLK6	Kallikrein related peptidase 6	Q92876	HPA019525
LDHA	Lactate dehydrogenase A	P00338	HPA075026
LHFPL3	LHFPL tetraspan subfamily member 3	Q86UP9	HPA077221
LRRTM4	Leucine rich repeat transmembran neuronal 4	Q86VH4	HPA061911
LRTM2	Leucine rich repeats and transmembraine domains 2	Q8N967	HPA062745
MAPT	Microtubule associated protein tau	P10636	HPA069570
MAPT	Microtubule associated protein tau	P10636	HPA069524
MASP1	Mannan binding lectin serine peptidase 1	P48740	HPA001617
MASP2	Mannan binding lectin serine peptidase 2	O00187	HPA029314
MASP2	Mannan binding lectin serine peptidase 2	O00187	HPA029313
MBP	Myelin basic protein	P02686	HPA049222
MBP	Myelin basic protein	P02686	HPA073581
MCHR2	Melanin concentrating hormone receptor 2	Q969V1	HPA050708

MEPE	Matrix extracellular phosphoglycoprotein	Q9NQ76	HPA038004
MEPE	Matrix extracellular phosphoglycoprotein	Q9NQ76	HPA071946
MMP9	Matrix metallopeptidase 9	P14780	HPA001238
MMP9	Matrix metallopeptidase 9	P14780	HPA063909
MOG	Myelin oligodendrocyte glycoprotein	Q16653	HPA021873
NCAN	Neurocan	O14594	HPA036814
NCAN	Neurocan	O14594	HPA077060
NEFH	Neurofilament heavy	P12036	HPA061615
NEFL	Neurofilament light	P07196	HPA014850
NETO1	Neuropilin and tolloid like 1	Q8TDF5	HPA073068
NEUROD2	Neuronal differentiation 2	Q15784	HPA049077
NEUROD6	Neuronal differentiation 6	Q96NK8	HPA074530
NKAIN2	Sodium/potassium transporting ATPase interacting 2	Q5VXU1	HPA035136
NSE	Neuron specific enolase	P09104	HPA070138
NSE	Neuron specific enolase	P09104	HPA078378
NRXN1	Neurexin 1	Q9ULB1	HPA059963
NTSR2	Neurotensin receptor 2	O95665	HPA007320
NTSR2	Neurotensin receptor 2	O95665	HPA077042
OLFM1	Olfactomedin 1	Q99784	HPA057444
OLIG1	Oligdendrocyte transcription factor 1	Q8TAK6	HPA077217
OLIG1	Oligodendrocyte transcription factor 1	Q8TAK6	HPA077730
OLIG2	Oligodendrocyte transcription factor 2	Q13516	HPA003254
OPALIN	Oligodendrocytic myelin paranodal and inner loop protein	Q96PE5	HPA014372
OPCML	Opioid binding protein/cell adhesion molecule like	Q14982	HPA065374
PACSIN1	Protein kinase C and casein kinase substrate in neurons 1	Q9BY11	HPA028852
PCDHA5	Protocadherin alpha 5	Q9Y5H7	HPA044557
PCDHGB1	Protocadherin gamma subfamily B 1	Q9Y5G3	HPA076182
PCDHGC5	Protocadherin gamma subfamily C 5	Q9Y5F6	HPA076140
PDYN	Prodynorphin	P01213	HPA049841
PDYN	Prodynorphin	P01213	HPA053342
PNMA2	PNMA family member 2	Q9UL42	HPA001936
POU3F2	Pou class 3 homeobox 2	P20265	HPA065187
PRRT2	Proline rich transmembrane protein 2	Q7Z6L0	HPA019203
PRRT2	Proline rich transmembrane protein 2	Q7Z6L0	HPA048045
PTPN5	Protein tyrosine phosphatase, non-receptor type 5	P54829	HPA031014
PTPRD	Protein tyrosine phosphatase, receptor type D	P23468	HPA054829
RASL10A	RAS like family 10 member A	Q92737	HPA056169
RPH3A	Rabphilin 3A	Q9Y2J0	HPA002475
RTN1	Reticulon 1	Q16799	HPA044249
S100B	S100 calcium binding protein B	P04271	HPA015768
SCN2A	Sodium voltage-gated channel alpha subunit 2	Q99250	HPA067350
SEPT_3	Septin 3	Q9UH03	HPA003548
SEZ6	Seizure related 6 homolog	Q53EL9	HPA012067
SLC12A5	Solute carrier family 12 member 5	Q9H2X9	HPA072058
SLC17A6	Solute carrier family 17 member 6	Q9P2U8	HPA039226
SLC17A7	Solute carrier family 17 member 7	Q9P2U7	HPA050458

SLC17A7	Solute carrier family 17 member 7	Q9P2U7	HPA063679
SLC1A2	Solute carrier family 1 member 2	P43004	HPA009172
SLC32A1	Solute carrier family 32 member 1	Q9H598	HPA058859
SLC32A1	Solute carrier family 32 member 1	Q9H598	HPA059985
SLC35F1	Solute carrier family 35 member F1	Q5T1Q4	HPA019576
SLC39A12	Solute carrier family 39 member 12	Q504Y0	HPA077034
SLC39A12	Solute carrier family 39 member 12	Q504Y0	HPA077354
SLC4A10	Solute carrier family 4 member 10	Q6U841	HPA034755
SLITRK1	SLIT and NTRK like family member 1	Q96PX8	HPA012414
SNCB	Synuclein beta	Q16143	HPA035876
SOX11	SRY-box 11	P35716	HPA000448
SPP1	Secreted phosphoprotein 1	P10451	HPA027541
SPTAN1	Spectrin alpha non-erythrocytic 1	Q13813	HPA007927
SPTBN1	Spectrin beta non-erythrocytic 1	Q01082	HPA013149
STMN4	Stathmin 4	Q9H169	HPA078407
STX3	Syntaxin 3	Q13277	HPA069176
SV2A	Synaptic vesicle glycoprotein 2A	Q7L0J3	HPA007863
SYN1	Synapsin 1	P17600	HPA000397
SYT1	Synaptotagmin 1	P21579	HPA064788
SYT11	Synaptotagmin 11	Q9BT88	HPA064091
TBR1	T-box brain 1	Q16650	HPA078657
TGFB1	Transforming growt factor beta 1	P01137	HPA047516
TGFB2	Transforming growth factor beta 2	P61812	HPA065065
TMEM132D	Transmembrane protein 132D	Q14C87	HPA010739
TMEM151A	Transmembrane protein 151A	Q8N4L1	HPA041035
TMEM59L	Transmembrane protein 59 like	Q9UK28	HPA010661
TNF	Tumor necrosis factor	P01375	HPA050631
TNF	Tumor necrosis factor	P01375	HPA077901
TNNI2	Troponin I2, fast skeletal type	P48788	HPA055938
TNNT2	Troponin T2 cardiac type	P45379	HPA017888
TPH1	Tryptophan hydroxylase 1	P17752	HPA022483
TRIM9	Tripartite motif contining 9	Q9C026	HPA067525
TTC9B	Tetratricopeptide repeat domain 9B	Q8N6N2	HPA042496
TUBB1	Tubulin beta 1 class VI	Q9H4B7	HPA043640
VCAM1	Vascular cell adhesion molecule 1	P19320	HPA069867
VEGFA	Vascular endothelial growth factor A	P15692	HPA069116
VEGFB	Vascular endothelial growth factor B	P49765	HPA059415
VEGFC	Vascular endothelial growth factor C	P49767	HPA004138
VEGFC	Vascular endothelial growth factor C	P49767	HPA073518
VSTM2B	V-set and transmembrane domain containing 2B	A6NLU5	HPA073612
VWC2L	Von Willebrand factor C domain containing protein 2 like	B2RUY7	HPA044815
ZDHHC22	Zinc finger DHHC-type containing 22	Q8N966	HPA062500

List of proteins included in the study and the corresponding Human Protein Atlas (HPA)* antibodies. Some proteins have two corresponding antibodies, but these do not necessarily yield the same results.
