# Supplementary material for: The cerebrospinal fluid proteome of preterm infants predicts neurodevelopmental outcome
Source: Front Pediatr. 2022 Jul 19;10:921444. doi: 10.3389/fped.2022.921444 (PMC9343678; doi:10.3389/fped.2022.921444)
Supplement: Supplementary file 2 [file Data_Sheet_2.PDF]

Supplementary Table 2
Protein level alterations in cerebrospinal fluid of preterm infants in correlation with culture results

Analyte	log_2 Fold Change	P
GDAP1L1	0.3997	0.0297
MEPE	0.5481	0.0297
FCN1	0.2034	0.0373
OPALIN	0.2028	0.0618
MAPT	0.2211	0.0661
AK5	0.1655	0.0708
VEGFA	0.1657	0.0708
C9	0.3867	0.0708
TGFB1	0.102	0.0757
IL6	0.1489	0.0863
AK5	0.2143	0.0921
GABRG1	0.1903	0.0922
SOX11	0.1846	0.0982
KCNF1	0.1807	0.0982
HTR2A	0.1479	0.1046
MASP2	0.1661	0.1046
ALDOC	0.1226	0.1046
HRH3	0.1264	0.1046
SLC17A6	0.1655	0.1113
GRM1	0.2977	0.1182
IL4	0.1938	0.1184
MCHR2	0.1679	0.1184
NR2E1	0.2055	0.1184
MMP9	0.1348	0.1184
ATP6V1G2	0.0927	0.1184
JPH3	0.1164	0.1184
LRRTM4	0.1104	0.1257
ALDOC	0.1692	0.1335
PNMA2	0.2009	0.1335
SPTAN1	0.2122	0.1335
TMEM59L	0.1408	0.1335
ERMN	0.1388	0.1335
POU3F2	0.234	0.1335
CACNG3	0.1652	0.1416
FCN1	0.2594	0.1501
MMP9	0.2312	0.1501
NEFL	0.1627	0.1501
PDYN	0.4797	0.1501
KCNV1	0.1027	0.1501
KCNA1	0.1375	0.1501
MEPE	0.1741	0.1682

CFI	0.1298	0.1683
VEGFC	0.1078	0.1683
GRM3	0.1453	0.1683
KCNJ9	0.1055	0.1683
IL4	0.1541	0.1683
C1orf61	0.1559	0.1683
CCL18	0.198	0.1778
HIF3A	0.1816	0.1778
PACSIN1	0.1712	0.1879
CFI	0.2319	0.188
MOG	0.3726	0.188
CLEC7A	0.1001	0.188
IFNG	0.1363	0.188
ZDHHC22	0.1164	0.188
BAALC	0.1237	0.188
IL10	0.1249	0.1983
HSPA4	0.2229	0.1984
PCDHA5	0.1284	0.209
SPTBN1	0.1165	0.2093
CAMK2G	0.1092	0.2093
NTSR2	0.1059	0.2093
SYN1	0.1296	0.2094
TNNT2	0.1902	0.2094
DSCAM	0.0992	0.2094
PDYN	0.2647	0.2094
TNF	0.1262	0.2094
CHRNB2	0.0692	0.2094
SLC17A7	0.0562	0.2094
SYT1	0.1561	0.2094
SCN2A	0.1223	0.2094
FEZF2	0.1168	0.2094
NEUROD6	0.1374	0.2094
KCNV1	0.1577	0.2094
FAM181B	0.181	0.2322
TTC9B	0.084	0.2323
LRTM2	0.1455	0.2323
GPM6B	0.1658	0.2324
CNTNAP4	0.181	0.2324
KCNC1	0.0765	0.2324
GRIN2A	0.0692	0.2324
NRXN1	0.1635	0.2324
GPR26	0.1536	0.2324
APP	0.222	0.2444
PTPN5	0.1041	0.2444
SLC32A1	0.0492	0.2445
TRIM9	0.1273	0.2571

NEFH	0.1431	0.2572
HIF1A	0.1364	0.2573
BCAN	-0.085	0.2573
ACVR1	0.3379	0.2573
TMEM132D	0.0889	0.2573
KCNF1	0.1252	0.2573
S100B	0.1992	0.2573
TPH1	0.1083	0.2573
TMEM151A	0.1817	0.2573
KCNC1	0.0879	0.2573
TNNI2	0.2033	0.2573
CASKIN1	0.1199	0.2573
GFAP	0.1508	0.2573
ENO2	0.1867	0.2573
CSPG5	0.1259	0.2573
OLFM1	0.1282	0.2702
HTR2C	0.0619	0.2837
GRIA2	0.0672	0.2838
CREG2	0.1794	0.2838
GALNT17	0.0783	0.2839
ERMN	0.1137	0.2839
MBP	0.2223	0.2839
LDHA	0.0807	0.2839
HIF3A	0.0656	0.2978
NEUROD2	0.1081	0.2978
KCNQ3	0.1532	0.2978
MBP	0.0785	0.2978
SLC39A12	0.068	0.2978
IL12A	0.0766	0.3122
SLC17A7	0.1083	0.3122
GABRA1	0.1742	0.3122
STX3	0.1494	0.3122
C11orf87	0.0398	0.3123
OPCML	0.1028	0.3123
CXCL1	0.1589	0.3123
AVP	0.1144	0.3123
C8orf46	0.0972	0.3123
IL1B	0.1416	0.3271
TNF	0.1103	0.3271
SLC4A10	0.1521	0.3424
APP	-0.1186	0.3425
SLC35F1	0.1915	0.3425
CAMK2G	0.112	0.3425
PTPRD	0.1227	0.3425
VEGFC	0.1564	0.3425
KLK6	0.1098	0.3582

GABRB2	0.0586	0.3582
OLIG1	0.1325	0.3746
LHFPL3	0.1789	0.3746
FCN2	0.0935	0.3746
STMN4	0.1338	0.3746
APC2	0.0984	0.3911
IL1B	0.0724	0.3912
GFAP	0.0459	0.4083
RPH3A	0.0532	0.4084
KIF5A	0.1003	0.4084
ARPP21	-0.1192	0.4084
BTBD17	0.144	0.4084
SLC32A1	-0.0007	0.4084
FRMPD4	0.1911	0.4084
C2orf80	0.0725	0.4084
ENO2	0.1072	0.4084
GABRA5	0.1307	0.4259
SLC12A5	0.0643	0.4439
SYT11	0.1129	0.444
ELAVL3	0.0211	0.444
ERC2	0.0886	0.4623
SLC1A2	0.1083	0.4812
VWC2L	0.0776	0.4813
RASL10A	0.0597	0.4813
MAPT	0.0263	0.4813
FCN3	0.1285	0.4813
SLITRK1	0.1465	0.5201
VEGFB	0.0699	0.5201
DLL3	0.0078	0.5201
TUBB1	0.1505	0.5202
GRIA2	0.0905	0.54
HPCA	0.0729	0.5402
C1QL2	0.0854	0.5402
MASP1	0.0513	0.5606
CACNG8	0.0599	0.5606
C9	0.5407	0.5607
SEPT_3	0.059	0.5607
CFB	0.0779	0.5607
BAALC	0.0563	0.5607
CNTNAP4	0.0422	0.5607
VCAM1	0.1159	0.5607
NETO1	0.0405	0.5607
HAPLN2	0.0307	0.5814
ACVR1C	0.1663	0.5815
OLIG2	0.1009	0.6026
OLIG1	0.0202	0.6026

C1QA	0.0882	0.6027
GPM6A	0.0805	0.6027
DIRAS2	0.0727	0.6027
CASKIN1	0.2611	0.6027
NCAN	-0.0803	0.6027
ACVR1	0.0616	0.646
FAM181B	0.0484	0.646
PCDHGB1	-0.0249	0.646
KIF3C	0.0296	0.6679
SV2A	0.0342	0.6906
GRIN1	-0.0053	0.6906
GRIN2A	0.0157	0.7363
C5	-0.1002	0.7363
TBR1	0.0344	0.7363
GAP43	-0.1178	0.7829
GPR37L1	0.0056	0.7829
SLC39A12	-0.1968	0.7829
SPP1	-0.0316	0.8303
NCAN	0.0371	0.8303
RTN1	-0.0795	0.8303
FABP7	0.0446	0.8303
NKAIN2	-0.0129	0.8542
NTSR2	-0.0262	0.8783
SEZ6	0.0915	0.8783
PRRT2	-0.0028	0.8783
SNCB	-0.0548	0.8783
PRRT2	-0.0365	0.8783
C1QB	0.0818	0.8783
CSPG5	0.0049	0.8783
VSTM2B	0.0051	0.8783
NPTX1	0.1008	0.8783
AQP4	-0.2254	0.9268
MASP2	-0.1338	0.9268
AMER2	-0.0294	0.9268
GPR37L1	-0.0091	0.9268
TGFB2	0.0098	0.9268
DSCAM	-0.2962	0.9268
GPM6B	0.0136	0.9268
AQP4	-0.295	0.9756
PCDHGC5	0.0848	0.9756
IL1A	0.0391	0.9756

Comparison of protein levels between preterm infants with and without positive cerebrospinal fluid/blood culture, n=16 and n=10, respectively.
