# Supplementary material for: The cerebrospinal fluid proteome of preterm infants predicts neurodevelopmental outcome
Source: Front Pediatr. 2022 Jul 19;10:921444. doi: 10.3389/fped.2022.921444 (PMC9343678; doi:10.3389/fped.2022.921444)
Supplement: Supplementary file 3 [file Data_Sheet_3.PDF]

Supplementary Table 3

Protein differences in term and preterm infants

Analyte	HPA number	log2 Fold Change	-logP	P*
CFB	HPA001817	0.9364	4.1418	7.21E-05
C5	HPA075945	0.7367	3.7025	1.98E-04
CASKIN1	HPA076882	1.8759	3.7025	1.98E-04
VCAM1	HPA069867	0.5288	3.2884	5.15E-04
ARPP21	HPA017303	0.473	3.1397	7.25E-04
MASP2	HPA029313	0.8159	3.0909	8.11E-04
ACVR1	HPA007505	0.4169	2.8996	1.26E-03
BCAN	HPA007865	0.3869	2.7145	1.93E-03
NETO1	HPA073068	0.4796	2.7145	1.93E-03
DSCAM	HPA074915	2.0084	2.7145	1.93E-03
ENO2	HPA078378	0.3362	2.6244	2.37E-03
MASP2	HPA029314	0.3163	2.4487	3.56E-03
APP	HPA001462	0.4503	2.3632	4.33E-03
FCN3	HPA071173	0.3392	2.2792	5.26E-03
PCDHGC5	HPA076140	0.2942	2.2792	5.26E-03
OLIG1	HPA077217	0.2258	2.2792	5.26E-03
MASP1	HPA001617	0.1379	2.1158	7.66E-03
SLC35F1	HPA019576	0.5329	2.0365	9.19E-03
C9	HPA029577	0.5303	1.8823	1.31E-02
TMEM132D	HPA010739	-0.4798	1.8075	1.56E-02
SPP1	HPA027541	0.2346	1.8075	1.56E-02
FABP7	HPA061703	0.2469	1.6625	2.18E-02
KLK6	HPA019525	0.2053	1.6271	2.36E-02
AVP	HPA071892	0.1438	1.6271	2.36E-02
AQP4	HPA014944	-0.4893	1.5922	2.56E-02
NKAIN2	HPA035136	0.1254	1.5576	2.77E-02
HAPLN2	HPA045765	0.106	1.5576	2.77E-02
AQP4	HPA014784	-0.5188	1.5234	3.00E-02
NPTX1	HPA077062	0.257	1.5234	3.00E-02
TBR1	HPA078657	0.0782	1.5234	3.00E-02
IL1A	HPA075911	0.1059	1.3904	4.07E-02
NTSR2	HPA007320	0.1349	1.3581	4.38E-02
SEPT_3	HPA003548	0.1191	1.3261	4.72E-02
MEPE	HPA071946	0.2976	1.3261	4.72E-02
VEGFC	HPA073518	0.11	1.3261	4.72E-02

The levels of 35 proteins differed in cerebrospinal fluid of term infants (n=10) and preterm infants with normal outcomes (n=13) at a threshold of p<0,05, established with Mann-Whitney U test. P* = <0,05. HPA; Human Protein Atlas
