# Supplementary material for: The cerebrospinal fluid proteome of preterm infants predicts neurodevelopmental outcome
Source: Front Pediatr. 2022 Jul 19;10:921444. doi: 10.3389/fped.2022.921444 (PMC9343678; doi:10.3389/fped.2022.921444)
Supplement: Supplementary file 4 [file Data_Sheet_4.PDF]

Supplementary Table 4

Protein differences in relation to outcome

Analyte	HPA number	log2 Fold Change	-logP	P*
SEZ6	HPA012067	0.5442	3.5325	2.93E-04
IL1A	HPA075911	0.2003	3.5325	2.93E-04
C11orf87	HPA034656	0.3363	3.4359	3.67E-04
VEGFC	HPA073518	0.3396	3.4359	3.67E-04
NKAIN2	HPA035136	0.156	3.3881	4.09E-04
HPCA	HPA043245	0.2219	3.2005	6.30E-04
TGFB2	HPA065065	0.1921	3.1544	7.01E-04
TBR1	HPA078657	0.1419	3.1544	7.01E-04
NCAN	HPA036814	0.414	3.1087	7.79E-04
GPM6B	HPA077843	0.1664	3.1087	7.79E-04
DIRAS2	HPA043758	0.2119	3.0634	8.64E-04
CNTNAP4	HPA057342	0.3431	2.9736	1.06E-03
SLC17A7	HPA050458	0.205	2.8853	1.30E-03
ELAVL3	HPA070436	0.2826	2.8853	1.30E-03
BCAN	HPA007865	0.3846	2.7983	1.59E-03
GPM6A	HPA017338	0.2804	2.7983	1.59E-03
CACNG8	HPA041351	0.2329	2.7983	1.59E-03
VWC2L	HPA044815	0.2348	2.7983	1.59E-03
VEGFB	HPA059415	0.2972	2.7983	1.59E-03
ERC2	HPA073739	0.2718	2.7983	1.59E-03
FAM181B	HPA075523	0.2111	2.7983	1.59E-03
APC2	HPA078002	0.2119	2.7983	1.59E-03
IL1B	HPA068737	0.1802	2.7127	1.94E-03
VEGFC	HPA004138	0.2184	2.6284	2.35E-03
BAALC	HPA027132	0.3563	2.6284	2.35E-03
PTPRD	HPA054829	0.2815	2.6284	2.35E-03
OPCML	HPA065374	0.3331	2.6284	2.35E-03
KCNQ3	HPA059375	0.2726	2.5868	2.59E-03
IL12A	HPA001886	0.2834	2.5455	2.85E-03
TMEM132D	HPA010739	0.2277	2.5455	2.85E-03
CAMK2G	HPA051785	0.2818	2.5455	2.85E-03
OLIG1	HPA077730	0.3293	2.5455	2.85E-03
NCAN	HPA077060	0.4988	2.5455	2.85E-03
SLC1A2	HPA009172	0.3488	2.464	3.44E-03
GPR26	HPA062736	0.2576	2.464	3.44E-03
FAM181B	HPA066861	0.2596	2.464	3.44E-03
LDHA	HPA075026	0.2205	2.464	3.44E-03
C2orf80	HPA078078	0.3051	2.464	3.44E-03
STMN4	HPA078407	0.2369	2.464	3.44E-03
CASKIN1	HPA055990	0.2686	2.3838	4.13E-03
DLL3	HPA060025	0.3278	2.3838	4.13E-03

NEUROD6	HPA074530	0.2373	2.3838	4.13E-03
C8orf46	HPA075134	0.2445	2.3838	4.13E-03
GRIA2	HPA008441	0.3265	2.3442	4.53E-03
TPH1	HPA022483	0.263	2.3049	4.96E-03
CNTNAP4	HPA031859	0.3331	2.3049	4.96E-03
IL4	HPA042270	0.2906	2.3049	4.96E-03
PRRT2	HPA048045	0.4695	2.3049	4.96E-03
SYT1	HPA064788	0.323	2.3049	4.96E-03
TRIM9	HPA067525	0.33	2.3049	4.96E-03
FEZF2	HPA068604	0.2137	2.3049	4.96E-03
GRIN1	HPA067773	0.3588	2.3049	4.96E-03
NPTX1	HPA077062	0.3022	2.3049	4.96E-03
APP	HPA031303	0.3254	2.266	5.42E-03
C1QL2	HPA057934	0.3505	2.266	5.42E-03
IL10	HPA071391	0.1584	2.266	5.42E-03
SYN1	HPA000397	0.2553	2.2274	5.92E-03
SV2A	HPA007863	0.3354	2.2274	5.92E-03
TNNT2	HPA017888	0.3393	2.2274	5.92E-03
PACSIN1	HPA028852	0.2623	2.2274	5.92E-03
SLC4A10	HPA034755	0.3484	2.2274	5.92E-03
HAPLN2	HPA045765	0.1629	2.2274	5.92E-03
KCNC1	HPA047634	0.275	2.2274	5.92E-03
GABRA1	HPA055746	0.2777	2.2274	5.92E-03
NRXN1	HPA059963	0.2351	2.2274	5.92E-03
NEFH	HPA061615	0.2418	2.2274	5.92E-03
CSPG5	HPA071779	0.545	2.2274	5.92E-03
PCDHGB1	HPA076182	0.1812	2.2274	5.92E-03
FCN2	HPA076099	0.3096	2.2274	5.92E-03
SLITRK1	HPA012414	0.2533	2.1513	7.06E-03
MEPE	HPA038004	0.3099	2.1513	7.06E-03
ERMN	HPA038295	0.2592	2.1513	7.06E-03
NR2E1	HPA055642	0.252	2.1513	7.06E-03
GRIA2	HPA070769	0.3621	2.1513	7.06E-03
VSTM2B	HPA073612	0.345	2.1513	7.06E-03
KIF3C	HPA075785	0.2962	2.1513	7.06E-03
CSPG5	HPA076601	0.2998	2.1513	7.06E-03
PTPN5	HPA031014	0.2326	2.1137	7.70E-03
OLFM1	HPA057444	0.2671	2.1137	7.70E-03
GABRB2	HPA067632	0.2994	2.1137	7.70E-03
SEPT_3	HPA003548	0.2072	2.0764	8.39E-03
KIF5A	HPA004469	0.2201	2.0764	8.39E-03
SPTBN1	HPA013149	0.2658	2.0764	8.39E-03
SLC32A1	HPA059985	0.193	2.0764	8.39E-03
SYT11	HPA064091	0.2709	2.0764	8.39E-03
POU3F2	HPA065187	0.4328	2.0764	8.39E-03
HIF3A	HPA074609	0.2567	2.0764	8.39E-03

CAMK2G	HPA051783	0.2764	2.0395	9.13E-03
SLC39A12	HPA077354	0.369	2.0395	9.13E-03
NTSR2	HPA007320	0.1899	2.0029	9.93E-03
PRRT2	HPA019128	0.1748	2.0029	9.93E-03
KCNC1	HPA041392	0.2558	2.0029	9.93E-03
CLEC7A	HPA050229	0.1952	2.0029	9.93E-03
GABRA5	HPA059644	0.3073	2.0029	9.93E-03
ALDOC	HPA067442	0.1834	2.0029	9.93E-03
KCNJ9	HPA070478	0.2176	2.0029	9.93E-03
KCNA1	HPA074471	0.2758	2.0029	9.93E-03
NEUROD2	HPA049077	0.252	1.9667	1.08E-02
GPM6B	HPA002913	0.3599	1.9308	1.17E-02
TMEM59L	HPA010661	0.3227	1.9308	1.17E-02
HTR2C	HPA052903	0.1561	1.9308	1.17E-02
ATP6V1G2	HPA068667	0.1125	1.9308	1.17E-02
OLIG1	HPA077217	0.1351	1.8952	1.27E-02
APP	HPA001462	0.4701	1.86	1.38E-02
NEFL	HPA014850	0.255	1.86	1.38E-02
ERMN	HPA038296	0.2418	1.86	1.38E-02
IFNG	HPA053530	0.203	1.86	1.38E-02
TNNI2	HPA055938	0.2857	1.86	1.38E-02
KCNF1	HPA062278	0.2608	1.86	1.38E-02
SLC17A7	HPA063679	0.2419	1.86	1.38E-02
JPH3	HPA076304	0.2059	1.86	1.38E-02
MASP1	HPA001617	0.1496	1.8251	1.50E-02
CFI	HPA024061	0.3844	1.7905	1.62E-02
HIF1A	HPA001275	0.301	1.7905	1.62E-02
RPH3A	HPA002475	0.0937	1.7905	1.62E-02
CHRNB2	HPA062865	0.108	1.7905	1.62E-02
NTSR2	HPA077042	0.1375	1.7905	1.62E-02
LRRTM4	HPA061911	0.1648	1.7562	1.75E-02
TMEM151A	HPA041035	0.3342	1.7223	1.90E-02
GRM3	HPA053434	0.2505	1.7223	1.90E-02
CCL18	HPA047485	0.3355	1.6887	2.05E-02
TNF	HPA077901	0.1473	1.6887	2.05E-02
HTR2A	HPA014011	0.2567	1.6554	2.21E-02
KCNF1	HPA014738	0.1661	1.6554	2.21E-02
DSCAM	HPA019324	0.3307	1.6554	2.21E-02
PCDHA5	HPA044557	0.355	1.6554	2.21E-02
IL1B	HPA064606	0.4306	1.6554	2.21E-02
VEGFA	HPA069116	0.2561	1.6554	2.21E-02
VCAM1	HPA069867	0.2079	1.6554	2.21E-02
LHFPL3	HPA077221	0.2739	1.6554	2.21E-02
ACVR1C	HPA007982	0.3091	1.5899	2.57E-02
AMER2	HPA039458	0.1567	1.5899	2.57E-02
HIF3A	HPA041141	0.1918	1.5899	2.57E-02

TGFB1	HPA047516	0.2072	1.5899	2.57E-02
RASL10A	HPA056169	0.2786	1.5899	2.57E-02
GFAP	HPA063513	0.2696	1.5899	2.57E-02
MAPT	HPA069570	0.2207	1.5899	2.57E-02
KCNV1	HPA069362	0.2324	1.5899	2.57E-02
AK5	HPA057255	0.2025	1.5576	2.77E-02
SPTAN1	HPA007927	0.2252	1.5257	2.98E-02
AK5	HPA019128	0.1715	1.5257	2.98E-02
FABP7	HPA061703	0.1	1.5257	2.98E-02
SOX11	HPA000448	0.223	1.494	3.21E-02
SLC17A6	HPA039226	0.1768	1.494	3.21E-02
MMP9	HPA001238	0.2177	1.4627	3.45E-02
GRIN2A	HPA004693	0.1693	1.4627	3.45E-02
GALNT17	HPA013624	0.29	1.4627	3.45E-02
ARPP21	HPA017303	0.3353	1.4627	3.45E-02
MBP	HPA049222	0.2933	1.4627	3.45E-02
ZDHHC22	HPA062500	0.1522	1.4627	3.45E-02
MMP9	HPA063909	0.2656	1.4627	3.45E-02
BAALC	HPA077738	0.3176	1.4627	3.45E-02
SLC32A1	HPA058859	0.1973	1.4317	3.70E-02
SLC12A5	HPA072058	0.2404	1.4317	3.70E-02
GRIN2A	HPA045139	0.1803	1.4011	3.97E-02
CREG2	HPA057596	0.2651	1.4011	3.97E-02
GDAP1L1	HPA063265	0.4206	1.4011	3.97E-02
SCN2A	HPA067350	0.1821	1.4011	3.97E-02
MEPE	HPA071946	0.5784	1.4011	3.97E-02
HSPA4	HPA010023	0.3393	1.3708	4.26E-02
FCN1	HPA001295	0.3036	1.3408	4.56E-02
PNMA2	HPA001936	0.1917	1.3408	4.56E-02
IL4	HPA070010	0.2087	1.3408	4.56E-02
GFAP	HPA055990	0.2249	1.3111	4.89E-02

Alterations in the levels of 164 proteins were observed at a threshold of $p < 0.05$. established with Mann-Whithney U test. HPA; Human Protein Atlas. P* = < 0.05.
